# Clinical parameters combined with radiomics features of PET/CT can predict recurrence in patients with high-risk pediatric neuroblastoma

**DOI:** 10.1186/s12880-022-00828-z

**Published:** 2022-05-28

**Authors:** Lijuan Feng, Luodan Qian, Shen Yang, Qinghua Ren, Shuxin Zhang, Hong Qin, Wei Wang, Chao Wang, Hui Zhang, Jigang Yang

**Affiliations:** 1 Department of Nuclear Medicine, Beijing Friendship Hospital, Capital Medical University, 95 Yong An Road, Xi Cheng District, Beijing, 100045 China; 2 Department of Surgical Oncology, National Center for Children’s Health, Beijing Children’s Hospital, Capital Medical University, Beijing, 100045 China; 3 Sinounion Medical Technology (Beijing) Co., Ltd. Beijing, 1 Yongtaizhuang North Road, Hai Dian District, Beijing, 100192 China; 4 Department of Biomedical Engineering, School of Medicine, Tsinghua University, Beijing, 100084 China

**Keywords:** Neuroblastoma, [^18^F]FDG, PET/CT, Radiomics, Recurrence

## Abstract

**Background:**

This retrospective study aimed to develop and validate a combined model based [^18^F]FDG PET/CT radiomics and clinical parameters for predicting recurrence in high-risk pediatric neuroblastoma patients.

**Methods:**

Eighty-four high-risk neuroblastoma patients were retrospectively enrolled and divided into training and test sets according to the ratio of 3:2. [^18^F]FDG PET/CT images of the tumor were segmented by 3D Slicer software and the radiomics features were extracted. The effective features were selected by the least absolute shrinkage and selection operator to construct the radiomics score (Rad_score). And the radiomics model (R_model) was constructed based on Rad_score for prediction of recurrence. Then, univariate and multivariate analyses were used to screen out the independent clinical risk parameters and construct the clinical model (C_model). A combined model (RC_model) was developed based on the Rad_score and independent clinical risk parameters and presented as radiomics nomogram. The performance of the above three models was assessed by the area under the receiver operating characteristic curve (AUC) and decision curve analysis (DCA).

**Results:**

Seven radiomics features were selected for building the R_model. The AUCs of the C_model in training and test sets were 0.744 (95% confidence interval [CI], 0.595–0.874) and 0.750 (95% CI, 0.577–0.904), respectively. The R_model yielded AUCs of 0.813 (95% CI, 0.685–0.916) and 0.869 (95% CI, 0.715–0.985) in the training and test sets, respectively. The RC_model demonstrated the largest AUCs of 0.889 (95% CI, 0.794–0.963) and 0.892 (95% CI, 0.758–0.992) in the training and test sets, respectively. DCA demonstrated that RC_model added more net benefits than either the C_model or the R_model for predicting recurrence in high-risk pediatric neuroblastoma.

**Conclusions:**

The combined model performed well for predicting recurrence in high-risk pediatric neuroblastoma, which can facilitate disease follow-up and management in clinical practice.

## Background

Neuroblastoma is one of the most common pediatric cancers, accounting for approximately 10% of all childhood malignant diseases [1]. Patients are stratified by age, stage, and molecular pathology into low-risk, intermediate-risk, and high-risk groups [2]. High-risk neuroblastoma requires systemic therapy (including induction chemotherapy, high-dose chemotherapy, and immunotherapy) and local therapy (surgery and radiotherapy). About 25% of high-risk neuroblastoma patients respond poorly to induction chemotherapy, may recurrence after initial therapy, and require alternative new treatment protocol before high-dose chemotherapy. These cases were considered refractory neuroblastoma patients. Other about 75% of high-risk patients may respond well initially, but recurrence before or after high-dose chemotherapy. These patients were considered recurrence patients [3]. Moreover, the 5-year event-free survival in high-risk patients was less than 50%. The survival ratio of patients with recurrence neuroblastoma is very low. Although the standard treatment protocol in these recurrence patients has not yet been established, multidisciplinary therapy is necessary [4]. Prevention of neuroblastoma recurrence is particularly difficult in high-risk patients. At present, neuroblastoma recurrence is diagnosed by imaging methods and cytological examinations [5]. However, tumor growth is generally advanced at this point. At this stage, the treatment protocol is very limited. Therefore, there is thus a great need to identify novel and effective biomarkers to predict neuroblastoma recurrence in these high-risk patients.

[^18^F]FDG PET/CT shows tumor glucose metabolic activity in the majority of neuroblastoma, though [^123^I]-metaiodobenzylguanidine (MIBG) scintigraphy remains the dominant disease-specific imaging method for this disease [6]. Currently, [^18^F]FDG PET/CT is selectively applied in some countries or centers because of MIBG availability, and [^18^F]FDG PET/CT is used for non-MIBG-avid neuroblastoma [7]. [^18^F]FDG PET/CT shows high per-patient sensitivity for lesion diagnosis in high-risk neuroblastoma patients [8].

Radiomics is a new quantitative imaging method that allows a thorough analysis of medical images data and attracted more and more attention in the field of medicine in recent years. Radiomics extracts numerous and quantitative information from medical images, including CT, MRI and PET, with high throughput to facilitate clinical decision-making [9]. The goals of radiomics are to improve decision support and reliability of prediction inexpensively and non-invasively [10]. Non-invasiveness is very crucial for pediatric patients. Radiomics also can provide a new angle for differential diagnosis, precision therapy, prediction of metastasis potential, therapy response [11] and prediction of tumor prognosis [12]. The value of radiomics based on [^18^F]FDG PET/CT in predicting recurrence has been demonstrated in previous studies [13–15]. However, radiomics based on [^18^F]FDG PET/CT in predicting recurrence in neuroblastoma, especially in the high-risk subgroups, has not been reported previously. Therefore, the present study investigated the value of radiomics based on [^18^F]FDG PET/CT in the prediction of recurrence of high-risk neuroblastoma patients. The final goal of the research aimed to develop and validate a combined model based [^18^F]FDG PET/CT radiomics and clinical parameters, which can predict the recurrence with good performance in high-risk neuroblastoma patients.

## Materials and methods

### Patients

The Institutional Review Board of Beijing Friendship Hospital, Capital Medical University approved this retrospective study and waived the requirement for written informed consent (Approval No.:2020-P2-091-02).

A total of 84 high-risk neuroblastoma patients were recruited between March 2018 and November 2019. The inclusion criteria of neuroblastoma were as follows: (1) pathologically confirmed neuroblastoma; (2) age ≤ 18 years at diagnosis; (3) complete [^18^F]FDG PET/CT imaging data; (4) complete clinical information; (5) without cancer-related therapy before PET/CT imaging; (6) complete laboratory and genetic data. High-risk neuroblastoma was defined according to (1) age older than 18 months and stage IV disease according to the International Neuroblastoma Staging System; or (2) any age and stage II–IV disease with MYCN amplification. All patients had received multidisciplinary treatment and then started maintenance treatment. These patients were monitored and evaluated throughout maintenance treatment, with follow-up ending on 31 October 2021. These patients were randomly divided into training and test sets with a ratio of 3:2.

### Determination of recurrence in high-risk neuroblastoma

Upon initial diagnosis, bone marrow biopsies and/or aspirates were performed, followed by microscopic examination to the identification of neuroblastoma cells. Serum levels of tumor markers [including neuron-specific enolase (NSE), serum ferritin and lactate dehydrogenase (LDH)] and urine vanillylmandelic acid (VMA) and homovanillic acid (HVA) were quantified. After multidisciplinary treatment, the therapeutic response was determined by quantification of serum tumor markers, urine VMA and HVA, microscopic examination of bone marrow, [^123^I]MIBG scanning, ultrasound, and computed tomography. Quantification of serum tumor markers, urine VMA and HVA, microscopic examination of bone marrow, and imaging tests were performed every 3 months, and [^123^I]MIBG scanning was performed every 6 months [1]. According to the Response Evaluation Criteria in Solid Tumors criteria, the response was classified as complete remission (CR), partial remission (PR), stable disease (SD), and progressive disease (PD). Patients with CR, PR, or SD received maintenance treatment. Patients with PD were considered as recurrence.

### [^18^F]FDG PET/CT imaging

All patients underwent PET/CT examinations on Siemens Biograph mCT-64 PET/CT following European Association of Nuclear Medicine guidelines [16, 17]. They were instructed to fast for at least 6 h and decrease intense exercises for at least 24 h before the examinations. 0.10–0.15MBq/kg of [^18^F]FDG was injected intravenously 40–60 min before the PET/CT scan. Firstly, the low-dose CT scan was performed for anatomical reference and attenuation correction, with 120 keV tube voltage and automatic modulated tube current. The CT image parameters were as follows: resolution 0.586 mm × 0.586 mm, 2 mm slice thickness, and matrix size 512 × 512. The PET scan was carried out with 2 min per bed position immediately after the whole-body CT scan. PET images were reconstructed using the ordered subsets-expectation maximization algorithm with time-of-flight. Attenuation corrections were applied during the reconstruction and a gaussian filter of 5 mm in full width at half-maximum was applied to the PET images. The PET image parameters were as follows: resolution 4.07 mm × 4.07 mm, 3 mm slice thickness, and matrix size 200 × 200.

**Fig. 1:**
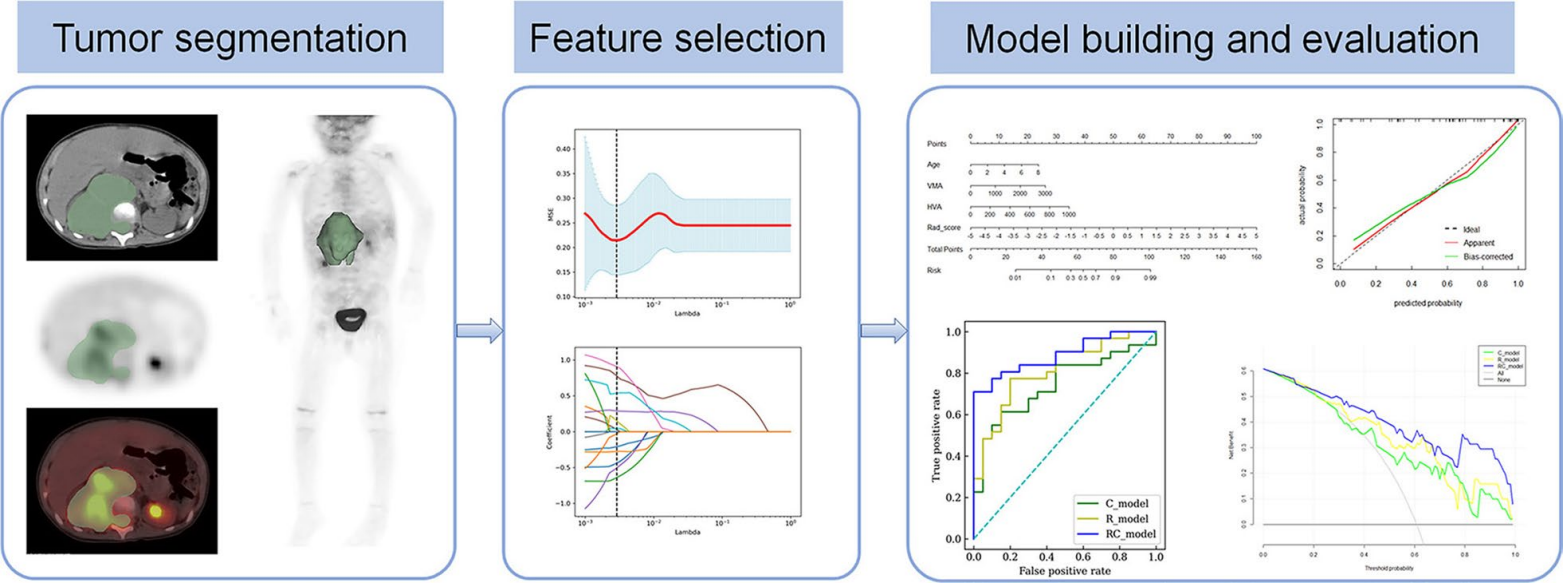
Workflow of the steps in our study. First, tumors were semi-automatically segmented by 3D Slicer. Second, radiomics features were extracted and selected by LASSO regression for further analysis. Finally, the combined model was developed based on the results of multivariate logistic regression in the training set, and the performance of the model was assessed by the ROC curve, calibration curve, and decision curve analysis

The regions of interest (ROI) of the primary tumor were semi-automatically segmented using 3D Slicer (version 4.10.1), which was delineated along the edge of neuroblastoma on CT images, including the entire tumor. The tumor size in our study was 1.7 cm–20 cm. The process may be variable and cause bias in the evaluation of derived radiomics features [18]. Therefore, the ROIs segmentation of each tumor was performed by two nuclear medicine physicians. To map the ROIs to the PET images, the PET images were resampled based on B-spline interpolation to ensure that they had the same voxel spacing as the CT images. Our study flow diagram is shown in Fig. 1.

### Feature extraction and selection

Radiomics features from CT and PET images were extracted separately using pyradiomics [19], an open-source python package for the extraction of radiomics features from medical imaging. At first, the PET/CT imaging and their ROIs were resampled based on linear interpolation to make them isotropic and improve the features’ repeatability. The voxel of the up-resampled PET imaging became 3 mm×3 mm×3 mm, and the voxel of CT imaging became 2 mm × 2 mm × 2 mm through down-resampling. PET and CT images were discretized by equal width bins with a standardized uptake value of 0.3 and 25 CT values (HU) [20, 21], respectively. First order features, shape features, gray level co-occurrence matrix (GLCM) features, gray level run length matrix (GLRLM) features, gray level size zone matrix (GLSZM) features, neighboring gray tone difference matrix (NGTDM) features, and gray level dependence matrix (GLDM) features were extracted from the original and the pre-processed images. The following methods were used in the imaging pre-processing: wavelet, square, square root, logarithm, exponential, and gradient filtering. The intraclass correlation coefficient (ICC) obtained based on the features extracted from the ROIs delineated by the two nuclear medicine physicians were used to assess the reliability of the variables, the features with ICC > 0.8 were maintained for further analysis. Then, the independent t-test or Mann-Whitney U test was used for univariate analysis, and the features with *P* < 0.05 were retained. Finally, the least absolute shrinkage and selection operator (LASSO) regression was applied for features selection and regularization in the training set.

### Model construction

The radiomics score (Rad_score) for each patient was calculated by using a linear combination of the selected features weighted by their respective coefficients, and the R_model was constructed based on Rad_score. The clinical parameters for predicting recurrence in high-risk neuroblastoma were screened by univariate and multivariate analysis and were selected as parameters to construct the C_model. RC_model was constructed by combining the Rad_score and the predictors in the C_model. All models were built and trained in the training set, and the prediction performance was evaluated in the training and test sets. The performance of each model was assessed by the area under the receiver operating characteristic (ROC) curve (AUC). The calibration of the RC_model was assessed with calibration curves. Decision curve analysis (DCA) was used to estimate the clinical utility of the RC_model, R_model, and C_model.

### Statistical analysis

Statistical analyses were performed with Python (version 3.7.8, www.python.org) and R (version 4.0.3, www.r-project.org). Univariate analysis was used to compare differences in the clinical parameters between the training and test sets, using the independent t-test or Mann-Whitney U test for quantitative data, and the chi-squared test for categorical variables. The Python package of “sklearn” was used for LASSO regression and the ROC curve. The nomogram and calibration curve were depicted using the “rms (R)” package. DCA was performed using the “rmda (R)” package. Two-sided *P* < 0.05 indicated statistical significance.

## Results

### Clinical parameters of patients

All clinical parameters of the patients between the training and test sets were compared, including gender, age, NSE, serum ferritin, LDH, urine VMA and HVA. The NSE, serum ferritin, LDH, urine VMA and HVA were acquired within two weeks before therapy. No significant difference was shown in all these clinical parameters between training and test sets (Table 1).

**Table 1:** Comparison of clinical parameters of the patients between the training and test sets

Factors	Training set	Test set	*P*
Gender			0.082
Female	30 (58.8%)	13 (39.4%)	
Male	21 (41.2%)	20 (60.6%)	
Recurrence			0.987
Yes	31 (60.8%)	20 (60.6%)	
No	20 (39.2%)	13 (39.4%)	
Age (years)	3.4 (1.9–4.7)	3.6 (2.8–5.3)	0.104
NSE (ng/mL)	297.6 (129.9–722.2)	511.0 (178.6–750.0)	0.357
Ferritin (ng/mL)	162.6 (69.9–351.5)	223.2 (118.3–342.2)	0.262
LDH (U/L)	605.0 (380.5–1114.0)	828.0 (545.0–1258.0)	0.234
VMA (µmol/L)	197.5 (57.4–674.6)	255.3 (87.5–620.2)	0.780
HVA (µmol/L)	52.9 (19.5–113.8)	91.0 (32.9–182.3)	0.189
*NSE* Neuron-specific enolase, *LDH* Lactate dehydrogenase, *VMA* Vanillylmandelic acid, *HVA* Homovanillic acid			

### Predictive model construction

A total of 2632 radiomics features were extracted from PET/CT images using pyradiomics. After assessing the robustness, 1016 out of 2632 features were retained for model building, with ICC > 0.8. Thirty-one features were retained after independent t-test or Mann-Whitney U test univariate analysis. Eventually, 7 features were extracted by LASSO regression. These 7 features (PET_original_gldm_LargeDependenceHighGrayLevelEmphasis, PET_wavelet-HLL_glcm_Imc1, PET_wavelet-LHL_firstorder_Median, PET_wavelet-HHH_glcm_Contrast, PET_wavelet-LLH_gldm_LowGrayLevelEmphasis, PET_wavelet-LLL_glcm_MCC, CT_wavelet-HLL_glszm_SmallAreaEmphasis) were used to build the R_model and calculate the Rad_score. Among these features, six were from PET images and one from CT images. The Rad_score for each patient was calculated by the following formula:

Rad_score = −0.97572 − 0.00042 × PET_original_gldm_LargeDependenceHighGrayLevelEmphasis + 20.38846 × PET_wavelet-HLL_glcm_Imc1 + 34.07874 × PET_wavelet-LHL_firstorder_Median + 59.78913 × PET_wavelet-HHH_glcm_Contrast − 15.62000 × PET_wavelet-LLH_gldm_LowGrayLevelEmphasis − 13.32169 × PET_wavelet-LLL_glcm_MCC − 6.07790 × CT_wavelet-HLL_glszm_SmallAreaEmphasis

**Fig. 2:**
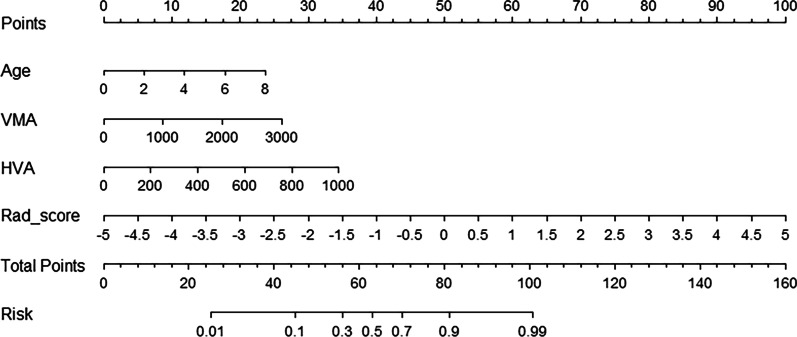
The radiomics nomogram incorporated age, VMA, HVA, and the Rad_score

All clinical parameters were compared between recurrence and non-recurrence groups (Table 2). Three clinical parameters (age, urine VMA and HVA) were then selected by multivariate analysis, which was used to construct the C_model. The RC_model was constructed by the three clinical parameters and Rad_score (Table 3). And the RC_model was presented as the nomogram based on the training set, which represented individualized prediction and visualized the proportion of each factor (Fig. 2).

**Table 2:** Comparison of clinical parameters and Rad_score of the patients between recurrence and non-recurrence group

Factors	Recurrence	Non-recurrence	*P*
Gender			0.066
Female	22 (43.1%)	21 (63.6%)	
Male	29 (56.9%)	12 (36.4%)	
Age (years)	4.0 (2.8–5.7)	2.9 (2.1–4.3)	0.041
NSE (ng/mL)	430.0 (213.1–782.8)	275.0 (100.0–674.0)	0.093
Ferritin (ng/mL)	255.0 (120.8–366.6)	123.7 (63.7–251.9)	0.017
LDH (U/L)	727.0 (521.5–1122.5)	605.0 (359.0–1435.0)	0.731
VMA (µmol/L)	537.0 (188.1–716.0)	78.6 (28.9–194.6)	< 0.001
HVA (µmol/L)	92.8 (32.6–182.3)	45.5 (13.9–59.2)	0.002
Rad_score	0.75 (0.21–1.76)	−0.83 (−1.27–0.03)	< 0.001
*HVA* Homovanillic acid, *LDH* Lactate dehydrogenase, *NSE* Neuron-specific enolase, *VMA* Vanillylmandelic acid

**Table 3:** Multivariate analysis of the factors used to build the RC_model

Parameters	OR (95% CI)	*P*
Rad_score	5.456 (1.693–17.586)	0.004
Age	1.654 (1.056–2.911)	0.045
VMA	1.004 (1.001–1.006)	0.017
HVA	1.004 (1.001–1.007)	0.013
*HVA* Homovanillic acid, *VMA* Vanillylmandelic acid, *OR* Odds ratio, *CI* Confidence interval

### Model performance

**Fig. 3:**
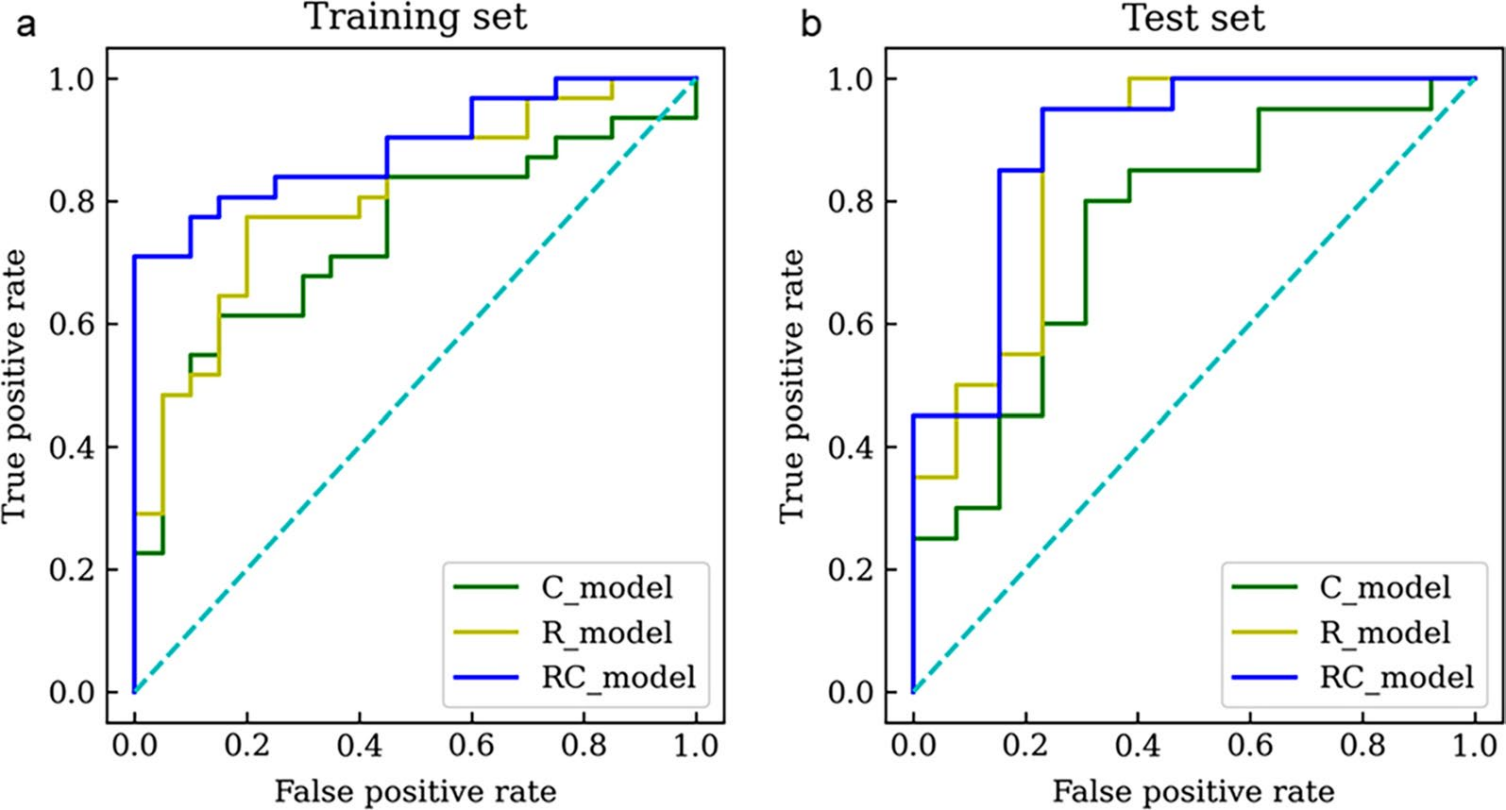
ROC curves for the RC_model, R_model and C_model in the training (**a**) and test sets (**b**)

**Fig. 4:**
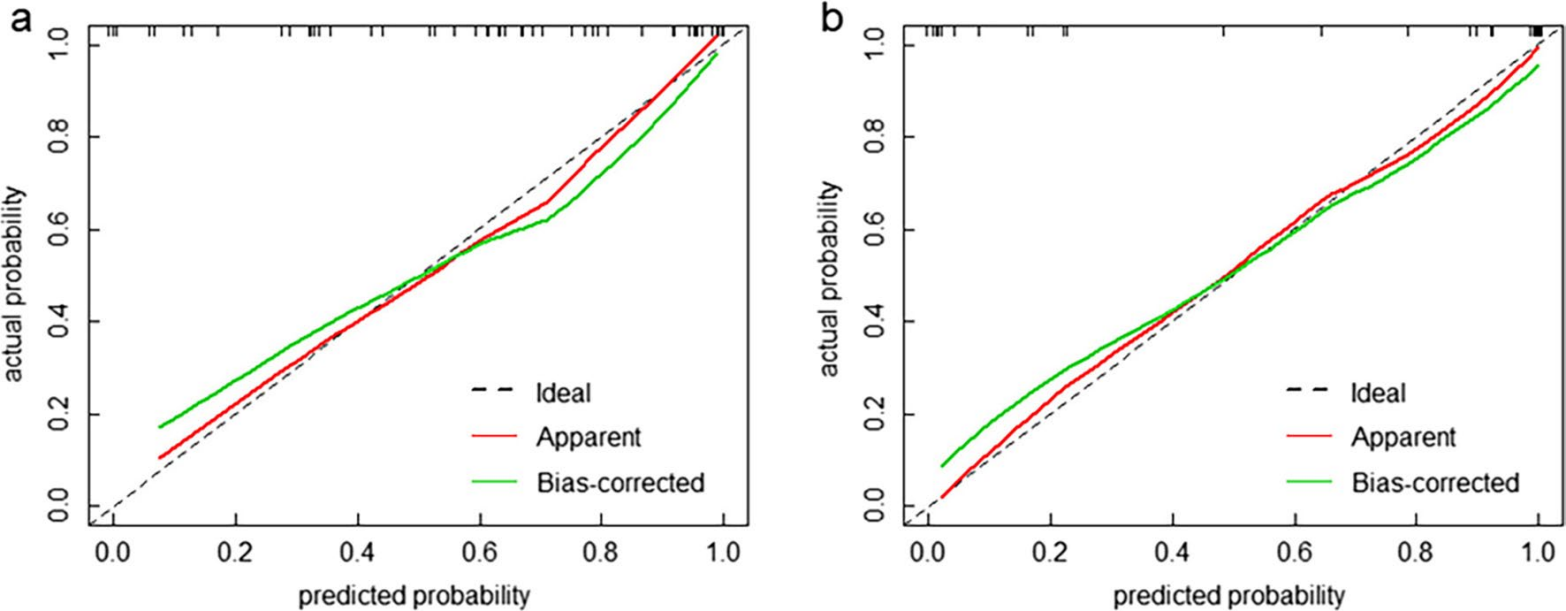
Calibration curves of the RC_model in the training (**a**) and test sets (**b**)

**Fig. 5:**
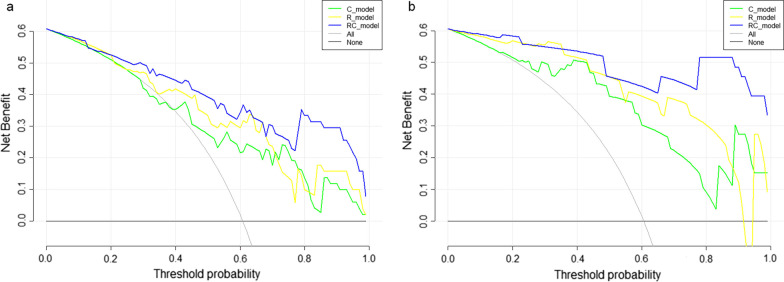
DCA for the RC_model, R_model and C_model in the training (**a**) and test sets (**b**)

The performance, including sensitivity, specificity, accuracy, and AUCs of different models were shown in Table 4. And the ROC curves of all models in both training and test sets were displayed in Fig. 3. The RC_model for recurrence prediction had the greatest performance in the training and test sets, with an AUC of 0.889 in the training set and an AUC of 0.892 in the test set. The calibration curves of the RC_model in the training and test sets were depicted in Fig. 4. It demonstrated that the RC_model has a good agreement in predicting recurrence in both the training and test sets. The DCA results for the RC_model, R_model, and C_model in the training and test sets were presented in Fig. 5. DCA showed that the RC_model added more net benefits for predicting recurrence in high-risk pediatric neuroblastoma than either the R_model or the C_model.

**Table 4:** Prediction performance of C_model, R_model, and RC_model in the training and test sets

Set	Model	Sensitivity (95%CI)	Specificity (95%CI)	Accuracy (95%CI)	AUC (95%CI)
Training	C_model	0.645 (0.454–0.808)	0.700 (0.457–0.881)	0.667 (0.521–0.792)	0.744 (0.595–0.874)
	R_model	0.774 (0.589–0.904)	0.700 (0.457–0.881)	0.745 (0.604–0.857)	0.813 (0.685–0.916)
	RC_model	0.806 (0.625–0.925)	0.800 (0.563–0.943)	0.804 (0.669–0.902)	0.889 (0.794–0.963)
Test	C_model	0.700 (0.457–0.881)	0.692 (0.386–0.909)	0.697 (0.513–0.844)	0.750 (0.577–0.904)
	R_model	0.800 (0.563–0.943)	0.769 (0.462–0.950)	0.788 (0.611–0.910)	0.869 (0.715–0.985)
	RC_model	0.900 (0.683–0.988)	0.769 (0.462–0.950)	0.848 (0.681–0.949)	0.892 (0.758–0.992)
*AUC* Area under the curve, *CI* Confidence interval

## Discussion

Although there are highly effective salvage therapies for patients with low-risk and intermediate-risk diseases who have a local recurrence, recurrence disease in high-risk neuroblastoma patients remains a clinical challenge. Therefore, accurate, non-invasive, and early recognition of high-risk neuroblastoma patients who had the possibility of recurrence is very important for clinical management. The present study demonstrated that the C_model and R_model had moderate power for predicting recurrence in the training and test sets. The RC_model showed a strong power for predicting recurrence in high-risk pediatric neuroblastoma, with sensitivity, specificity, accuracy, AUCs of 0.806, 0.800, 0.804, and 0.889 in the training set and 0.900, 0.769, 0.848, and 0.892 in the test set. The predictive ability of the RC_model was better than the C_model and R_model.

Among the clinical parameters, three clinical parameters were used to construct a prediction model, including age, urine VMA and HVA. Age is an important risk stratification or prognostic index in neuroblastoma. International Neuroblastoma Pathology Classification is defined by multiple factors, including age at diagnosis [22]. The risk stratification schema proposed by the Children’s Oncology Group is defined by several factors, including age, ploidy, histology, et al. [23]. VMA and HVA levels in urine, the serum levels of serum ferritin, NSE, and LDH are considered characteristic tumor markers of neuroblastoma. These parameters are helpful at the initial diagnosis, response assessment, and monitoring recurrence of neuroblastoma [24]. At diagnosis, abnormal results were found in 92% for VMA and HVA of urine. Abnormal results at recurrence or progression were demonstrated in 54% for HVA and VMA of urine. The sensitivity of these markers was higher for metastasis compared with local recurrence [24]. However, the predictive efficacy of the C_model is not very high with AUCs of 0.744 in the training set, and 0.750 in the test set.

The value of [^18^F]FDG PET/CT in high-risk neuroblastoma patients has been investigated previously. [^18^F]FDG PET/CT and bone marrow sampling may suffice for disease monitoring in high-risk neuroblastoma [25]. [^18^F]FDG PET/CT based on visual analysis has significant implications for prognostic assessment in these patients [8]. The maximum standardized uptake value representing the metabolic activity of the tumor was identified as poor prognostic factors associated with decreased survival. [^18^F]FDG uptake may assist in the identification of patients with poor prognoses [26]. However, the images of [^18^F]FDG PET/CT were analyzed visually and semi-quantitatively in above these studies. These analytical methods were influenced by many factors, including physician experience, interval time between [^18^F]FDG injection and scan, blood glucose serum, and so on. Therefore, more objective and accurate analytical methods of [^18^F]FDG PET/CT images were needed in neuroblastoma patients, especially in high-risk sub-group patients. Radiomics transforms medical images into quantitative indexes through high-throughput extraction by data-assessment algorithms for predicting important clinical outcomes [27]. After this research, the R_model showed a moderate power for predicting recurrence, with AUCs of 0.813 in the training set and 0.869 in the test set. Seven radiomics features were selected for R_model construction including six wavelet features. Wavelet transform was applied to feature extraction given the fact that wavelet transform-based features showed good capability in tumor classification and prognosis. The original image was decomposed into eight categories by performing wavelet transformation on three axes. On each axis, the signal of the image was decomposed into the high-frequency and low-frequency components by the wavelet transform, including “LLL_,” “LLH_,” “LHL,” “LHH_,” “HLL_,” “HLH_,” “HHL_,” and “HHH_”. The wavelet-based features are generally considered useful for radiomics studies [28, 29]. In the present study, one GLDM feature was used to build a predictive model. GLDM as one of the texture features quantifies gray level dependencies in an image, which is defined as the number of connected voxels within a specific distance that is dependent on the center voxel. Texture can reveal tumor heterogeneity, which is relevant to the underlying biology, and radiomics analysis provides a feasible method to unlock the buried information beyond the perception of the human eyes [30].

In the present study, to achieve a more holistic model, we incorporated clinical parameters with radiomics features, leading to a significantly improved prediction efficacy of the RC_model than the C_model and R_model for predicting recurrence in high-risk neuroblastoma patients. This finding is noteworthy because the predictive efficacy of the C_model and R_model is moderate. To minimize potential limitations of a R_model including overfitting and poor reliability, we applied the standardized image prepossessing and multi-step feature selection to build the R_model with nonredundant, reliable, and informative features. Additionally, DCA demonstrated the RC_model added more net benefits for predicting recurrence in high-risk pediatric neuroblastoma than either the R_model or the C_model.

The potential clinical significance of the present study included: (1) RC_model based on radiomics features and clinical parameters provides an accurate method in a non-invasive way for predicting recurrence of high-risk neuroblastoma; (2) The early prediction of recurrence in high-risk neuroblastoma can help clinicians following-up these patients, guiding further management in these sub-group patients.

This study had several limitations. Firstly, this was a retrospective study with relatively small sample size, particularly in the test set, which might have selection bias and influence the robustness and generalizability of our predictive model. And we randomly divided the patients into training and test sets in the ratio of 3:2, which may lead to the poor stability of our results. We will try other methods, such as using the k-fold cross-validation strategy to divide the training and test sets to improve the stability of our results in future studies. Secondly, to ensure better accuracy, we semi-automatically delineated the primary tumor, however, it was labor-intensive, time-consuming, and subject to inter-and intra-observer variability. Moreover, it produced poorly reproducible results. Therefore, in future studies, we will try to overcome these issues using automatic segmentation methods to improve the reproducibility of radiomics studies. And we segmented the primary tumor on the CT images only, anatomical information often does not match metabolic information at all though we resampled PET images based on B-spline interpolation to ensure that they had the same voxel spacing as the CT images. Therefore, we will try to use a proper method to segment the ROI on PET images in future studies, and segment both CT and PET images to make our results more accurate. Finally, we only performed internal validation. Therefore, it is necessary to validate the results in large, multicentric cohorts and improve the reliability of models for predicting the recurrence of high-risk neuroblastoma.

## Conclusions

Our combined model integrating clinical parameters with the Rad_score from [^18^F]FDG PET/CT images could predict recurrence in high-risk neuroblastoma with high discriminatory ability. The predictive model could serve as a potential decision support tool for both clinicians and radiologists, and help guide appropriate management for high-risk neuroblastoma patients.

## Data Availability

The data presented in this study are available on request from the corresponding author.
